# Shall I Work with Them? A Knowledge Graph-Based Approach for Predicting Future Research Collaborations

**DOI:** 10.3390/e23060664

**Published:** 2021-05-25

**Authors:** Nikos Kanakaris, Nikolaos Giarelis, Ilias Siachos, Nikos Karacapilidis

**Affiliations:** Industrial Management and Information Systems Lab, MEAD, University of Patras, 26504 Rio Patras, Greece; giarelis@ceid.upatras.gr (N.G.); ilias.siachos@upnet.gr (I.S.); karacap@upatras.gr (N.K.)

**Keywords:** knowledge graph, link prediction, natural language processing, document representation, future research collaborations, graph kernels, word embeddings

## Abstract

We consider the prediction of future research collaborations as a link prediction problem applied on a scientific knowledge graph. To the best of our knowledge, this is the first work on the prediction of future research collaborations that combines structural and textual information of a scientific knowledge graph through a purposeful integration of graph algorithms and natural language processing techniques. Our work: (i) investigates whether the integration of unstructured textual data into a single knowledge graph affects the performance of a link prediction model, (ii) studies the effect of previously proposed graph kernels based approaches on the performance of an ML model, as far as the link prediction problem is concerned, and (iii) proposes a three-phase pipeline that enables the exploitation of structural and textual information, as well as of pre-trained word embeddings. We benchmark the proposed approach against classical link prediction algorithms using accuracy, recall, and precision as our performance metrics. Finally, we empirically test our approach through various feature combinations with respect to the link prediction problem. Our experimentations with the new COVID-19 Open Research Dataset demonstrate a significant improvement of the abovementioned performance metrics in the prediction of future research collaborations.

## 1. Introduction

The development of knowledge graph-based approaches for predicting future research collaborations has received increasing attention in recent years [1,2]. In these approaches, a scientific article that has been written by two researchers denotes implicitly a collaboration between them [3]. In general, the majority of the existing knowledge graph based approaches builds on concepts and methods from graph theory to infer knowledge that is not explicitly provided, exploiting the structural characteristics of the corresponding research graph [4]. However, these approaches ignore the extremely useful (but unstructured) textual data that are in most cases available in documents such as scientific articles and reports [5]; as a consequence, they are not able to meaningfully incorporate both structural and textual information into their knowledge graph.

Aiming to overcome the above issues, this paper proposes the development and deployment of a novel scientific knowledge graph that enables the co-existence and joint utilization of structured as well as unstructured data, such as author, document and word nodes. Expanding on past work, the documents of a scientific graph are represented as a graph of documents (graph-of-docs approach) [6,7,8], which facilitates the discovery of future research collaborations by using prominent link prediction algorithms. In addition, recent advances in graph mining enable our approach to calculate the similarity between two graph-of-docs representations, using graph similarity techniques (e.g., graph kernels and graph neural networks). For the implementation of our approach, we use the Neo4j graph database (https://neo4j.com, accessed on 25 April 2021) as far as the implementation and storage of the proposed knowledge graph is concerned, the Python programming language for coding purposes, and the TensorFlow and scikit-learn Machine Learning (ML) libraries for the training of our prediction models.

To evaluate the approach described in this paper, we benchmark it against different well-established combinations of graph-related measures. For the ML models proposed, we include the accuracy, precision, and recall metrics as they are considered the most robust and popular metrics for performance assessment. In the experiments included in this work, the COVID-19 Open Research Dataset (CORD-19) is utilized. Having in mind that the size of the dataset could affect our approach in a variety of ways (e.g., overfits or underfits), we consider ten (10) different datasets, extracted from the original dataset. The experimental results lead to a significant improvement of the link prediction accuracy, compared to the ML models considered. The final version of code, datasets, and evaluation results of our work are openly accessible on GitHub (https://github.com/nkanak/cordkel, accessed on 25 April 2021). The main contributions of this paper are the following:

- We investigate whether the integration of unstructured textual data into a single knowledge graph affects the performance of a link prediction model.
- We study the effect of previously proposed graph kernels-based approaches on the performance of an ML model, as far as the link prediction problem is concerned.
- We propose a three-phase pipeline that enables the exploitation of structural and textual information, as well as of pre-trained word embeddings.
- We empirically test our approach through various feature combinations with respect to the link prediction problem.

To the best of our knowledge, this is the first work on the prediction of future research collaborations that combines structural and textual information of a scientific knowledge graph through a purposeful integration of graph algorithms and natural language processing (NLP) techniques. The remainder of this paper is organized as follows. Background concepts and related work are introduced in Section 2. In Section 3, our approach is presented in a thorough and elaborated fashion, while the experiments carried out to evaluate our approach are reported in Section 4. The novelty, future work directions as well as the limitations of the proposed approach are discussed in Section 5.

## 2. Background and Related Work

Our approach in predicting future research collaborations makes use of a set of graph theory, graph-based text representation, NLP, and knowledge graph techniques.

### 2.1. Graph Related Concepts

In graph theory, many different graph types have been proposed, which vary upon the types of nodes and edges, the features they may share and their overall structure [9]. Bellow, we present graph concepts and notations that are used in the context of this paper.

**Definition** **1** **(Graph).** *Let a graph* G=(V,E) *be defined as a tuple consisting of a set of vertices (or nodes)* V *and a set of edges* E ⊆V×V*, which connects a pair of vertices. The size of the graph is defined by the number of edges* |V|, *while the number of the vertices is defined as* |E|.

**Definition** **2** **(Directed** **Graph).** *A directed graph is a graph* G=(V,E)*, where the edges are directed by arrows. Directed graphs are also known as digraphs.*

**Definition** **3** **(Weighted** **Graph).** *A weighted graph is a graph*  G=(V,E), *in which a function* w:E→ℝ *assigns edges with some weights or numbers.*

**Definition** **4** **(Labeled** **Graph).** *A labeled graph is a graph* G=(V,E), *in which a function* ℓ:V∪E→Σ *assigns labels to its vertices and edges from a discrete set of values* Σ*. Depending on the application at hand, there may exist a necessity to label only the vertices, in which case the graph is called node-labeled graph. Accordingly, if only the edges are labeled, the graph is called edge-labeled graph. Finally, a graph with both vertices and edges labelled is called fully-labeled.*

**Definition** **5** **(Attributed** **Graph).** *A graph* G=(V,E) *is an attributed graph if there exists a function* $f:V\cup E\to {\mathbb{R}}^{d}$ *that assigns real-valued vectors to its vertices and edges.*

**Definition** **6** **(Adjacency** **Matrix).** *Adjacency matrix offers a systematic mapping of the graph structure. Let* $A_{ij}$ *be the element in the* $i^{th}$ *row and* $j^{th}$ *column of the matrix A. Then, the adjacency matrix A can be defined as:*

$$A_{ij}\;=\;\{\begin{array}{ll} 1, & if\;\left\{v_{i},v_{j}\right\}\in E\\ 0, & otherwise \end{array}$$

**Definition** **7** **(Degree** **Matrix).** *Given a graph* G=(V,E), *the degree matrix is defined as the diagonal matrix* $D=diag\left(d_{ii}\right)$ *where:*

$$d_{ii}=\sum_{j}A_{ij}$$

*is the number of neighbors for each node of the graph.*

**Definition** **8** **(Walk,** **Path).** *A walk in a graph* G=(V,E) *is a series of vertices* $v_{1},\;v_{2},\ldots ,v_{k+1}$ *where* $v_{i}\in V$ *for all* 1≤i≤k+1 *and edges* $\left\{v_{i},\;v_{i+1}\right\}\in E$ *for all* 1≤i≤k*. The number of the edges in this sequence is called the length of the walk. If all the vertices in the walk are different from each other, the walk is called a path.*

**Definition** **9** **(Shortest** **Path).** *A path from node* $v_{i}$ *to node* $v_{j}$ *is defined as the shortest path between these two nodes, if there exist no other path between these two nodes with smaller length.*

**Definition** **10** **(Graph** **Isomorphism).** *A graph isomorphism between two labeled/attributed graphs* $G_{i}=\left(V_{i},E_{i}\right)$ *and* $G_{j}=\left(V_{j},E_{j}\right)$ *is a bijection* $\phi :V_{i}\to V_{j}$, *that preserves adjacencies, i.e.,* $\forall v,u\in V_{i}:\left(v,u\right)\in E_{i}\Leftrightarrow \left(\phi \left(v\right),\phi \left(u\right)\right)\in E_{j}$, *and labels, i.e.,* *if* $\psi \in V_{i}\times V_{i}\to V_{j}\times V_{j}$ *is the mapping of vertex pairs implicated by the bijection ϕ such that* ψ((v,u))=(ϕ(v),ϕ(u)), *then*, *the conditions* $\forall v\in V_{i}:\ell \left(v\right)\equiv \ell \left(\phi \left(v\right)\right)$ *and* $\forall e\in E_{j}:\ell \left(e\right)\equiv \ell \left(\psi \left(e\right)\right)$ *must hold*, *where* ≡ *denotes that two labels are considered equivalent.*

### 2.2. Graph Measures and Indices

A variety of graph measures and indices which extract knowledge based on the structural characteristics of a graph have been proposed in the literature [10]. Below, a small subset of them is presented, which are applied in the approach described in this paper.

The Common Neighbors measure for two nodes *a* and *b*, denoted by *CN(a*, *b)*, calculates the number of common neighbor nodes (i.e., nodes that are connected with both *a* and *b*) [11]. It is defined as:

CN(a, b)=|Ν(a) ∩ Ν(b)|

where *Ν(x)* denotes the set of neighbors for a node *x*.

The Total Neighbors measure for two nodes *a* and *b*, denoted by *TN(a*, *b)*, takes into consideration all neighbors of these two nodes (contrary to the Common Neighbors measure which deals with only the neighbor nodes). It is defined as:

TN(a, b)=|N(a) ∪ N(b)|

The Preferential Attachment measure for a pair of nodes *a* and *b*, denoted by *PA(a*, *b)*, is defined as the product of the in-degree values of the two nodes [12]. The assumption behind this measure is that the likelihood of two nodes being connected in the future is far greater for two highly connected nodes, in contrast to two loosely connected ones. This measure is defined as:

PA(a, b)=|N(a)| × |N(b)|

The Adamic Adar measure for two nodes *a* and *b*, denoted by *AA(a*, *b)*, calculates the sum of the inverse logarithm of the degree of the neighbor nodes shared by the nodes *a* and *b* [13]. This measure assumes that the likelihood of a neighbor node to be influential in the future depends on how low its degree may be. It is defined as:

$$AA\left(a,\;b\right)=\sum_{c\;\epsilon \;N\left(a\right)\cap N\left(b\right)}\left(\frac{1}{\log\left|c\right|}\right)$$

Last but not least, the Jaccard Coefficient index for two nodes *a* and *b*, denoted by *J(a*, *b)*, extends the CN measure mentioned above, differentiating slightly by considering the amount of the intersection of their neighbor nodes over the union of them [14]. It is defined as:

$$J\left(a,\;b\right)=\frac{\left|N\left(a\right)\;\cap \;N\left(b\right)\right|}{\left|N\left(a\right)\;\cup \;N\left(b\right)\right|}$$

### 2.3. Graph Kernels

Kernels have gained much attention as a generalization technique in machine learning applications, aiming to model and compute the similarity among objects. Specifically, graph kernels have proven to be the dominant approach for learning on graph-structured data [15]. Machine learning tasks with graph-based data are directly related to graph comparison, which presents many complex difficulties, as the nature of graph-structured data tends to be very different from the usual representations (vectors, matrices etc.). To overcome these problems, graph kernels capture the semantics and the latent characteristics that are inherent in the graph in a computationally efficient time, thus achieving state-of-the-art results on several datasets.

Graph kernels compute the similarity between two graphs, based on the common substructures they share. In the literature, a variety of substructures have been proposed, including random walks [16,17], shortest paths [18] and subtrees [19]. Generally, we can express a graph kernel as the inner product defined in some Hilbert space, e.g., given a kernel *k*, we can define a mapping function φ: G→H into a Hilbert space H such that $k\left(G_{1},\;G_{2}\right)=\langle \varphi \left(G_{1}\right),\;\varphi (G_{2})\rangle$ for all graphs $G_{1},G_{2}\in G$. Depending on the substructures in hand, graph kernels achieve to compute the similarity among graphs with respect to these substructures. Therefore, for each application under consideration, one must carefully choose which kernels should be utilized.

Depending solely on substructures of graphs, however, can create the downside of ignoring global structure and characteristics, which may be valuable. For graphs with small size, such local approaches may be appropriate; however, for larger graphs, graph kernels may fail to perform equivalently well. As a result, the problem of incorporating global properties of graphs to graph kernels has gained some attraction and a recent work attempts to tackle this issue [20]. We refer to [15] for an in-depth review of graph kernels.

#### 2.3.1. Pyramid Match Graph Kernel

The pyramid match graph kernel makes use of mappings of nodes of a graph to a low-dimensional vector space (embeddings) [20]. The most profound way of producing node embeddings is by using the eigenvectors of the *d* largest in magnitude eigenvalues of the adjacency matrix *A* of the graph. By creating a *d*-dimensional vector for each node, we define a *d*-dimensional hyperplane (hypercube), where each node embedding is represented as a point. Then, the kernel partitions the hypercube into regions of increasingly larger size and for each region takes a weighted sum of the matches, i.e., sum all the co-occurrences of points into the same region. As the regions expand, the matches are less and less weighted.

More specifically, the kernel repeatedly fits a grid with cells of increasing size to the *d*-dimensional hypercube. Each cell is related only to a specific dimension and its size along that dimension is doubled at each iteration, whereas it remains constant for the rest of the dimensions. Given a sequence of iterations (levels) from *0* to *R*, at level *r* the d-dimensional kernel region has $2^{r}$ cells along each dimension and $D=2^{r}\cdot d$ cells in total. Then, given two graphs $G_{i},G_{j}$, let $H_{G_{i}}^{r},\;H_{G_{j}}^{r}$ denote the histograms of $G_{i}$ and $G_{j}$ at iteration *r*, and $H_{G_{i}}^{r}\left(k\right),\;H_{G_{j}}^{r}\left(k\right)$ the number of nodes that lie in the $k^{th}$ cell for each graph. The number of nodes (matches) found in the same region at iteration *r* is computed as follows:

$$I\left(H_{G_{i}}^{r},H_{G_{j}}^{r}\right)=\overset{D}{\underset{k=1}{\sum }}\min\left(H_{G_{i}}^{r}\left(k\right),H_{G_{j}}^{r}\left(k\right)\right)\;$$

However, there is no need to compute the matches found in every iteration as a large number of these matches should have been computed in a previous iteration. Instead, we want to compute the number of new matches found at each level which is given by $I\left(H_{G_{i}}^{r},H_{G_{j}}^{r}\right)-I\left(H_{G_{i}}^{r+1},H_{G_{j}}^{r+1}\right)\;$for *r = 0*, *…*, *R − 1*. Then, the pyramid match kernel is defined as follows:

$$k\left(G_{i},G_{j}\right)=I\left(H_{G_{i}}^{R},H_{G_{j}}^{R}\right)+\overset{R-1}{\underset{r=0}{\sum }}\frac{1}{2^{R-r}}\left(I\left(H_{G_{i}}^{r},H_{G_{j}}^{r}\right)-I\left(H_{G_{i}}^{r+1},H_{G_{j}}^{r+1}\right)\right)$$

where $\frac{1}{2^{R-r}}$ is the decreasing weight appointed to each level.

#### 2.3.2. Propagation Kernel

The basic idea behind the propagation kernels is the propagation of label information between nodes of the graph, based on the overall graph structure. The general framework was introduced in [21], where the graph is considered to have attributes on nodes (attributed graph). Propagation kernel defines a probability distribution *P* of size |V|×d, where *d* is the size of attributes in the feature space. This probability distribution is then updated for a given number of iterations $t_{MAX}$ by concatenating the neighbors’ attributes, on the basis of the following substitution rule:

$$P^{t+1}\to D^{-1}\cdot A\cdot P^{t}$$

where *D* is the degree matrix and *A* is the adjacency matrix for a graph. Then, the probability matrix *P* is utilized to bin nodes, through a hash function producing a hash vector $\varphi \left(G^{t}\right)$. Finally, we compute the kernel $K\left(G_{i}^{t},G_{j}^{t}\right)$ at iteration *t* between two graphs $G_{i},G_{j}$ as follows:

$$K\left(G_{i}^{t},G_{j}^{t}\right)=\underset{v_{i}\in V_{i}}{\sum }\underset{v_{j}\in V_{j}}{\sum }k\left(v_{i},v_{j}\right)\;=\;\langle \varphi \left(G_{i}^{t}\right),\varphi \left(G_{j}^{t}\right)\rangle$$

This procedure is repeated for $t_{MAX}$ iterations, until the kernel converges to a local minimum.

### 2.4. Graph-Based Text Representations

In the graph-of-words textual representation [22] each document of a corpus is represented as a single graph. Specifically, every unique word of a document is depicted as a graph node and the co-occurrence between two words (i.e., if two words appear simultaneously within a sliding window of text) is denoted with an edge connecting the corresponding nodes. As far as the size of the sliding window is concerned, it is suggested in Rousseau et al. [23] that a window of four words seems to be the most appropriate value, as the impact on the performance and the accuracy of the ML models is negligible. Taking into consideration the co-occurrence between terms results in feature engineering being more sophisticated in the graph-of-words representation, compared to the bag-of-words one. Nevertheless, the limitations of the graph-of-words text representation are that: (i) the importance of a word for a whole set of documents cannot be assessed; (ii) it is not possible for multiple documents to be represented in a single graph, and (iii) the expendability of the representation in order to support more complex data architectures is not intuitive.

To address the drawbacks of the graph-of-words representation, the authors in [7] have proposed the graph-of-docs representation, where multiple textual documents are depicted in a single graph. In this way: (i) the investigation of the importance of a term into a whole corpus of documents is easily calculated, and (ii) the co-existence of heterogeneous nodes in the same graph renders the representation easily expandable and adaptable to more complicated data. In this paper, the graph-of-docs model is utilized to represent the textual data of a knowledge graph.

### 2.5. Word Embeddings

In general, word embeddings map a corpus of words into a vector space where similar words have similar vector representations. A list of techniques for learning word embeddings has been proposed in the literature [24], the most popular ones including *Word2Vec* [25], *GloVe* [26] and *fastText* [27]. Word embeddings are broadly used in many NLP tasks ranging from text classification and sentiment analysis to more sophisticated ones such as spam detection and question-answering. They improve the accuracy of an ML model, prevent overfitting and assist in terms of generalization [28,29]. In majority, word embeddings are trained on large datasets, which are usually language or domain specific. Hence, they enable the learning techniques to capture statistical correlations between the words and, subsequently, produce word embeddings for a specific NLP task (e.g., finding cars similar to a Jaguar or finding animals similar to a jaguar). A common practice is to start with pre-trained word embeddings and adjust them to a specific domain or NLP task. Popular pre-trained word embeddings include *GoogleNews* using Word2Vec and *Common Crawl* using GloVe. We refer to [30] for an in-depth review of the available pre-trained word embeddings. It is also noted that the majority of the techniques for learning word embeddings has been expanded to serve the learning of sentence, paragraph and document embeddings [31,32]. For instance, *Doc2Vec* [33] relies on Word2Vec to represent each document of a corpus as a vector.

### 2.6. Predicting Future Research Collaborations

As far as link prediction techniques for the discovery of future research collaborations are concerned, works closest to ours are proposed in [34,35,36,37,38]. In particular, in [34], the authors rely only on a co-authors network’s topology aspects, and the proximity of a pair of nodes in order to predict future research collaborations between them. In [35], the authors recommend an approach, where structural properties are used to calculate the probability of future research collaborations in heterogeneous bibliographic networks, with varying types of nodes (e.g., papers, authors, venues, topics) and edges (e.g., publish, write, mention, cite, contain). They leverage the multiple relationships among the papers to improve the overall accuracy of their link prediction algorithm.

In [36], the authors predict potential research collaborations by combining link prediction techniques with a random forest classifier. For each pair of nodes of a co-authorship network, a wide range of topology-based measures such as Adamic Adar and Common Neighbors, are calculated and, then, combined with location-based characteristics related to the authors. Hence, the position of the authors in the co-authorship network and their location are used to generate future collaboration proposals. In [37], the authors construct a co-authorship network that represents research collaborations from 1980 to 2005 in the field of computer science. In this case, a variety of graph theory algorithms and classical statistical techniques are employed in order to extract the co-authorship network properties. The dataset used is composed of 451,305 papers from 283,174 authors.

In [38], the authors use medical co-authorship networks along with link prediction algorithms to predict future research collaborations. For a given author, potential collaborators are identified, as long as they complement her skillset. For each pair of author nodes common topological and structural measures are extracted, such as Adamic Adar, Common Neighbors and Preferential Attachment, and multiple ML models are utilized for the prediction of possible future collaborations.

Adopting a broader link prediction perspective, additional works (e.g., [39,40,41]) describe the task of predicting possible relationship types between nodes of a particular network from various and interesting scopes (such as social networks and friendship suggestions).

## 3. The Proposed Approach

**Figure 1:**
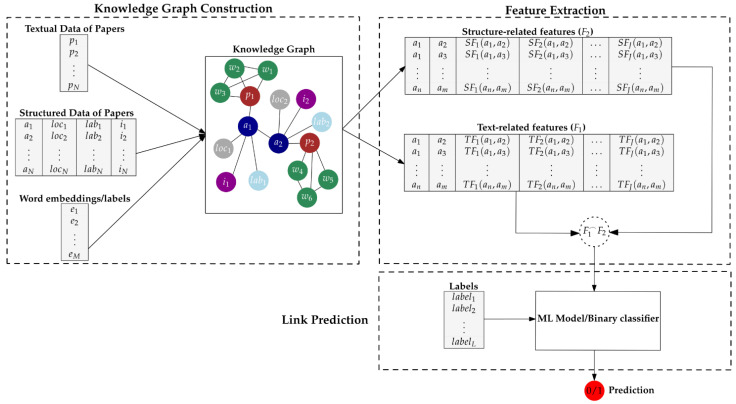
The phases of the proposed approach. *p_x_* denotes nodes of the ‘*Paper*’ type. *w_x_* denotes nodes of the ‘*Word*’ type. *a_x_* denotes nodes of the ‘*Author*’ type. *loc_x_* denotes nodes of the ‘*Location*’ type. *i_x_* denotes nodes of the ‘*Institution*’ type. *lab_x_* denotes nodes of the ‘*Laboratory*’ type. The word embedding of a word (*w_x_*) is denoted by *e_x_*. *SF_x_* and *TF_x_* denote structure-related and text-related features, respectively. *label_x_* denotes the label (0 or 1) that corresponds to the sample *x* of the given dataset. $\hat{F_{1}F_{2}}$ denotes the concatenation of the structure-related and text-related features, aiming to generate the feature vector of the sample *x* of the given dataset.

We first compose a scientific knowledge graph containing both structured and unstructured textual data (Section 3.1). The unstructured textual data are integrated into the knowledge graph via graph-of-docs text representation (see Section 2.4). Then, a series of graph measures (see Section 2.2) and graph kernels (see Section 2.3) are employed for feature extraction associated to both textual and structural information concerning the entities of the knowledge graph (Section 3.2). Finally, we map the whole problem of predicting future research collaborations to a link prediction task, by utilizing the features produced in the previous step for the deployment of an ML model (Section 3.2). To sum up, our approach is divided in three phases, namely Knowledge Graph Construction, Feature Extraction and Link Prediction. Figure 1 illustrates the phases of the proposed approach.

### 3.1. Knowledge Graph Construction

**Figure 2:**
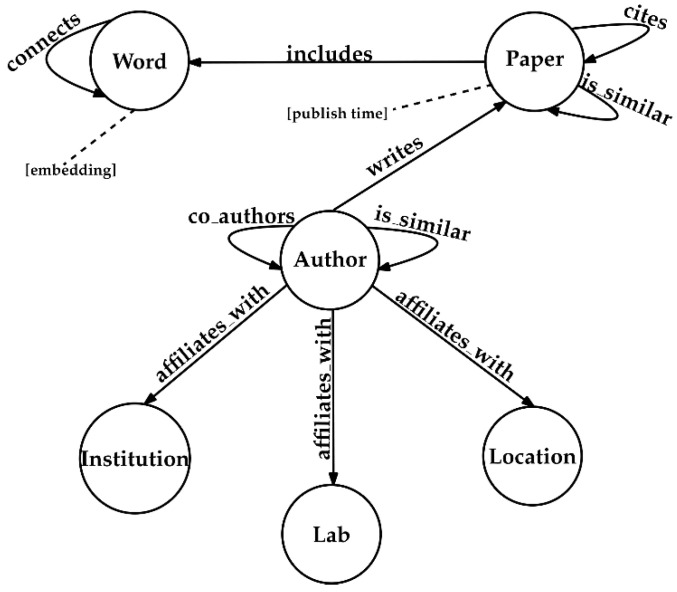
The data schema of the proposed scientific knowledge graph. Dotted lines connect properties associated with the entities of the knowledge graph.

The nature of knowledge graphs enables the co-existence of multiple types of entities and relationships in the same data schema. Specifically, our scientific knowledge graph includes entity nodes with types such as ‘*Paper*’, ‘*Author*’, ‘*Laboratory*’, ‘*Location*’, ‘*Institution*’ and ‘*Word*’, as well as types of relationship edges such as ‘*is_similar*’, ‘*cites*’, ‘*writes*’, ‘*includes*’, ‘*connects*’, ‘*co_authors*’ and ‘*affiliates_with*’ (see Figure 2).

As far as entity nodes are concerned, nodes that are labeled as ‘*Paper*’ express scientific documents; the ‘*Author*’ entity represents an author of a scientific paper or document the laboratory of an author and its location are represented as a ‘Laboratory’ and ‘Location’ entity, respectively; each ‘*Institution*’ entity corresponds to the institution of an author; finally, for the representation of a unique word of a scientific paper a ‘*Word*’ entity is utilized.

With respect to relationship edges, the existence of a specific word to a certain paper is depicted with the ‘*includes*’ relationship, which connects a ‘*Paper*’ with a ‘*Word*’ entity. Similarly, the co-occurrence of a pair of words within a predefined sliding text window is modeled through a ‘*connects*’ relationship, which is only applicable between two ‘*Word*’ entities. The graph-of-docs representation of the textual data of the available papers can be defined as the subgraph constructed by the ‘*Word*’ and ‘*Paper*’ entities, as well as the ‘*includes*’, ‘*connects*’ and ‘*is_similar*’ relationships

Furthermore, the ‘*is_similar*’ relationship links pairs of ‘*Paper*’ or ‘*Author*’ nodes. In the former case, it denotes the similarity between two papers represented as graph-of-docs. In the latter, it denotes the similarity between the documents associated to the two authors. We can produce an author similarity subgraph by considering the subgraph that is composed of the ‘*Author*’ entities and the ‘*is_similar*’ relationships.

Finally, the citation of a paper from another one is depicted as a ‘*cites*’ relationship between two ‘*Paper*’ nodes; the writer of a scientific document is represented with a ‘*writes*’ relationship between an ‘*Author*’ with a ‘*Paper*’ entity; accordingly, the relationship of an author with an institution and its location and laboratories are depicted with an ‘*affiliates_with*’ relationship connecting an ‘*Author*’ entity with an ‘*Institution*’, ‘*Location*’ or ‘*Laboratory*’ entity, respectively; The ‘*co_authors*’ relationship denotes a research collaboration between the connected ‘*Author*’ entities. Similarly to the the graph-of-docs and authors similarity subgraphs, we can construct a co-authors’ subgraph, by isolating the available ‘*Author*’ entities and the ‘*co_authors*’ relationships from the original scientific knowledge graph.

The produced knowledge graph enables the user to gain insights about a variety of tasks through the employment of well-studied graph algorithms. Such tasks would be recommending similar research work, finding nearby experts based on the ‘*Location*’ entities, and discovering future research collaborations; this paper focuses on the last of these tasks.

### 3.2. Feature Extraction

The proposed knowledge graph enables the extraction of informative features for each entity. Each feature of an entity encapsulates either structural or textual information related to the whole knowledge graph; in this paper, we concentrate on the extraction of features that describe the relationship between two ‘*Author*’ nodes. To extract structure-related features, we can use graph measures or indices such as common neighbors, preferential attachment and Adamic Adar (see Section 2.2). To extract text-related features, we can use graph similarity techniques including graph neural networks and graph kernels (see Section 2.3); for the experiments reported in this paper, we employ graph kernels. It is also noted here that text-related features can be further enriched, either by attaching labels or word embeddings to the ‘*Word*’ nodes of each paper of the knowledge graph (see Section 2.5). Finally, for the generation of the feature vector of each sample of a given dataset, we concatenate the structure-related and text-related feature vectors (Figure 1).

### 3.3. Link Prediction

For the discovery of future research collaborations, a wide range of link prediction and ML techniques are employed. Specifically, in our approach, the issue of suggesting future research collaborations is reduced to the common binary classification problem. Such an approach enables us to construct a link prediction algorithm by predicting the presence (or absence) of a ‘*co_authors*’ relationship for a pair of ‘*Author*’ entities. Various binary classifiers, including logistic regression, k-nearest neighbors, linear support vector machines, decision tree, and neural networks have been considered in the related literature [42]. In the experiments reported in this paper, we have used the logistic regression and neural network ones.

## 4. Experimental Evaluation

To implement and evaluate our approach, we utilized the Python programming language, the TensorFlow [43] and scikit-learn [44] ML libraries, as well as the *GraKeL* [45] library for graph kernels. In addition, we used the word embeddings from the *CORD-19 Swivel text embedding* module of the TensorFlow Hub (available online: https://tfhub.dev/tensorflow/cord-19/swivel-128d/3, accessed on 25 April 2021). Furthermore, we utilized the Neo4j database (https://neo4j.com, accessed on 25 April 2021), aiming to store the instance of the graph-of-docs representation and the knowledge graph. All the material related to our experiments and the evaluation can be also found at https://github.com/nkanak/cordkel (accessed on 25 April 2021).

### 4.1. Evaluation Metrics

To evaluate our approach, we use three widely used metrics, namely *accuracy*, *precision* and *recall*. These metrics rely on the number of true positives (|*TP*|), true negatives (|*TN*|), false positives (|*FP*|), and false negatives (|*FN*|) predictions of each classification model. In particular, accuracy is the percentage of correct predictions. Recall estimates the ability of the classifier to find all the positive samples. Precision estimates the ability of the classifier not to label as positive a negative sample. The accuracy metric is defined as:

$$accuracy=\frac{\left|TP\right|+\left|TN\right|}{\left|TP\right|+\left|TN\right|+\left|FP\right|+\left|FN\right|}$$

The precision metric is defined as:

$$precision=\frac{\left|TP\right|}{\left|TP\right|+\left|FP\right|}$$

The recall metric is defined as:

$$recall=\frac{\left|TP\right|}{\left|TP\right|+\left|FN\right|}$$

To further evaluate our approach, we also take into account the values of the *average cross-entropy* metric, which we utilize as a loss function, whenever it is possible. As opposed to the accuracy, precision and recall metrics, cross-entropy is not affected by the order of the training data. In particular, binary cross-entropy measures the dissimilarity between the distribution of the real value y and the predicted value $\hat{y}$ of a model. The binary cross-entropy is defined as follows:

$$H\left(y,\hat{y}\right)=-y\cdot \log\left(\hat{y}\right)-\left(1-y\right)\cdot \log\left(1-\hat{y}\right)$$

Accordingly, we can define the average cross-entropy among *N* samples as:

$$J=\frac{1}{N}\overset{N}{\underset{n=1}{\sum }}H\left(y_{n},\hat{y_{n}}\right)=-\frac{1}{N}\;\overset{N}{\underset{n=1}{\sum }}y_{n}\cdot \log\left(\hat{y_{n}}\right)+\left(1-y_{n}\right)\cdot \log\left(1-\hat{y_{n}}\right)$$

Finally, we use the micro sign test to investigate whether there is any statistical significance in improvement (*p* < 0.05) of each metric between the baselines and the proposed models.

### 4.2. The CORD-19 Dataset

**Figure 3:**
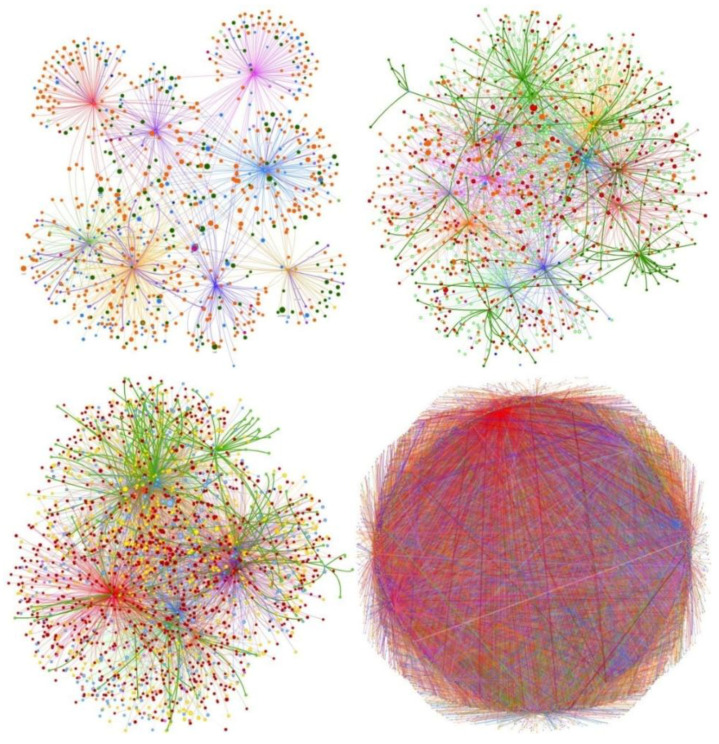
Snapshots of the knowledge graph that is generated from the CORD-19 dataset: limited to 1000 (**upper-left**), 2000 (**upper-right**), 3000 (**bottom-left**) and 30,000 (**bottom-right**) nodes. The different node and edge colors highlight the heterogeneity of the produced graph.

The COVID-19 Open Research Dataset (CORD-19) [46] includes 63,000 scientific articles, related to COVID-19 and other coronaviruses. It is available from the Allen Institute for AI and Semantic Scholar. The scientific papers in CORD-19 have been retrieved from several famous medical-related repositories, such as PubMed Central (PMC), bioRxiv, Elsevier, World Health Organization (WHO) and medRxiv. Each scientific paper in CORD-19 is associated with an array of attributes, including ‘*publish time*’, ‘*citations*’, ‘*title*’, ‘*abstract*’ and ‘*authors*’. Most of the scientific papers (i.e., 51,000) also has a ‘*full text*’ attribute. It is apparent that this dataset benefits the research community as far as the COVID-19 related research is concerned. However, due to the unstructured nature of the textual data included in the dataset, many shortcomings exist in terms of gaining useful insights. As already mentioned in the literature, by combining a graph-based text representation and a knowledge graph, we are able to generate a semi-structured representation of the data, which alleviates the existing drawbacks [4,5,47]. To construct the scientific knowledge graph, we use the ‘*publish time*’, ‘*abstract*’ and ‘*authors*’ attributes of the scientific papers. Due to hardware limitations, we do not utilize the *full text* of each scientific paper. Figure 3 illustrates different snapshots of the knowledge graph that corresponds to the CORD-19 dataset. The different node and edge colors highlight the heterogeneity of the produced graph. For illustration purposes, we limit the depicted nodes to 1000, 2000, 3000 and, respectively. As Figure 3 shows, the number of relations between the nodes increases radically in proportion to the number of nodes.

#### Generation of Datasets for Predicting Future Research Collaborations

We generate ten (10) new balanced datasets using the CORD-19, aiming to investigate whether our approach performs well, irrespective of the properties of a dataset. To create a sample of each dataset, we use the ‘*authors*’ similarity subgraph an the ‘*co_authors*’ subgraph, which is composed of the ‘*co_authors*’ edges. We note that edges also have as a property the first collaboration year of two authors.

Table 1 reports the number of samples of the training and testing subset for each one of the produced datasets. The samples of each dataset have been randomly chosen. Table 2 describes the features of each sample of the dataset. We conduct a list of experiments using different combinations of features, aiming to investigate whether the performance of a ML model is affected by the features under consideration (Table 3). We note that each training sample is selected from an earlier instance of the ‘*co_authors*’ subgraph, which is generated from the ‘*co_authors*’ edges that first appeared within or before the year 2013. Accordingly, the testing subset incorporates ‘*co_authors*’ edges generated after 2013. We separate the dataset by using the time to ensure that any data leakage is avoided among the testing and the training subset [34].

### 4.3. Baseline Feature Combinations

We benchmark the feature combinations extracted by our approach against three baseline combinations, namely AA_J, AA and J (see Table 3 for a detailed explanation):

- **AA_J** [8]: It combines the structural information of the knowledge graph using the Adamic Adar measure as well as the textual similarity of the papers of two authors using the Jaccard index.
- **AA** [13]: It utilizes the structural information of the knowledge graph using the Adamic Adar measure.
- **J** [14,38]: It utilizes the textual similarity of the papers of two authors using the Jaccard index.

### 4.4. Evaluation Results

The evaluation of our approach is achieved by assessing the sensitivity of the ML models towards the various feature combinations, regarding the binary classification problem. The binary classifiers used in our experiments are logistic regression (LR) and neural network (NN); for the training of the NN model, we use five epochs, the Adam optimizer, two hidden layers with 50 and 25 units, respectively, while we utilize the average binary cross-entropy as a loss function. Further experimentations with a plethora of classifiers and various hyperparameter configurations is available online: https://github.com/nkanak/cordkel (accessed on 25 April 2021). As already mentioned in Section 4.1, our performance metrics include the accuracy, precision and recall of the binary classifiers. In addition, we test for statistical significance against the AA_J feature combination, since it has the best performance across all the baseline combinations. The experimental results are summarized in Table 4 and Table 5.

The obtained results indicate that in many cases (e.g., AA_PM or AA_WPM) the inclusion of a text-related feature increases the average accuracy, precision and recall scores, while still remaining unaffected by the nature of the binary classifier. Additionally, it is worth mentioning that our feature combinations correctly predict the majority of the future collaborations, by observing the average precision score compared to the average recall score. The best average accuracy and recall scores are achieved by the LR classifier, using the AA_WPM feature combination. Thus, by combining these two features we get the optimal outcome. The best average precision score is achieved by both classifiers, using the J feature combination. However, by using the J feature combination, both classifiers have a low recall score, which means that they always classify all the samples of a test dataset as candidate future collaborations, something that is totally wrong.

In addition, it is revealed that features including ‘*total_neighbors*’, ‘*preferential_attachment*’ and ‘*common_neighbors*’ (appearing in the ALL feature combination) add noise to the LR classifier, but not to the NN classifier; this is due to the fact that NNs are able to capture more complex and non-linear connections between the given features and the predicted values than LR models. Specifically, we note that LR models are built on the assumption that the independent variables are linearly related to the log of probabilities of features. However, in most real-world applications, this relationship tends to be non-linear, thus making LR models behave poorly. Moreover, logistic regression requires average or no collinearity between features, which may not always be the case (e.g., ‘*common_neighbors*’ and ‘*jaccard*’ features may be highly correlated). On the other hand, LR models offer greater interpretability, as we can estimate the importance of the features by examining the corresponding model coefficients. Such a feature importance estimation can lead to fine-tuned systems, by iteratively disregarding less important features until a local optimum is reached.

**Figure 4:**
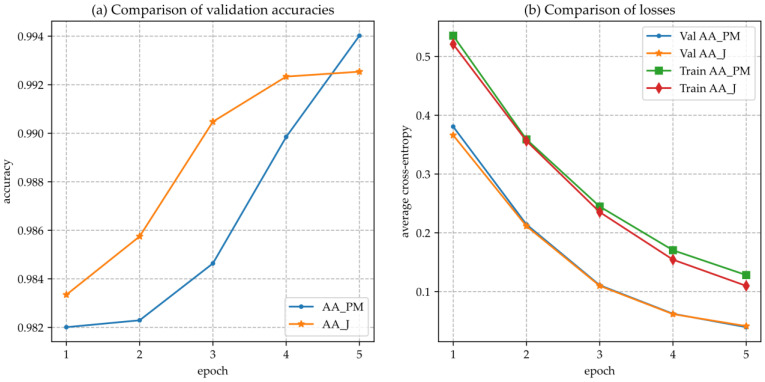
(**a**) Comparison of validation accuracies of the NN model using the AA_PM and the AA_J feature combinations; (**b**) Comparison of cross-entropy of the NN model using the AA_PM and the AA_J feature combinations.

It is also observed that metrics or kernels for calculating textual similarities, which incorporate into their formula the terms with their corresponding attributes as well as the structure of the text (e.g., WPM), perform better compared to metrics that deal only with the absence or the presence of a term (e.g., J). By considering the abovementioned remarks, we conclude that using both textual and structural characteristics of a scientific knowledge graph leads to more accurate ML models and less prone to overfitting. Moreover, the NNs are optimized towards minimizing average binary cross-entropy, which can lead to more dynamic and robust models as cross-entropy measures information content and is not as much sensitive to the order of the training data as accuracy-based metrics (see Figure 4).

## 5. Concluding Remarks

In this paper, we apply link prediction techniques on scientific knowledge graphs for recommending future research collaborations. Our three-phase approach incorporates both structured and unstructured textual data in every knowledge graph. To do so, we leverage the graph-of-docs text representation. For our experiments, we construct ten datasets using the CORD-19 dataset. We benchmark our approach against several feature combinations. Our approach demonstrates a significant improvement of the average accuracy, precision and recall of the task of predicting future collaborations, while, also, minimizing the average cross-entropy, as far as the NN models are concerned.

The proposed approach diverges from the state-of-the-art ones since it takes into consideration both the textual similarity of the papers of each set of authors as well as the associated structural information. The approaches that deal solely with unstructured textual data rely on NLP methods and text representations, which require the construction of huge feature spaces. As a result, the ‘curse-of-dimensionality’ phenomenon re-emerges.

Key enablers that drive the development of the proposed approach are the availability of large computing power, the existence of big volumes of unstructured textual and social network data (e.g., data from a research graph), as well as the availability of a range of well-tried and powerful ML software libraries for analyzing complex graph-structured data [48,49]. Building on a meaningful and flexible combination of textual and structural information related to an author and his papers, our approach enables ML models to better identify future research collaborations. Simply put, our approach improves significantly the quality of the prediction, while enabling ML models to interpret more complex data patterns. However, diverse problems and limitations still exist; these refer to: (i) the time to construct the knowledge graph, (ii) the ability of the graph-based models to add new nodes (e.g., author or paper nodes) to an existing scientific knowledge graph, and (iii) the performance of the models on graphs with rich node attribute information (e.g., nodes having high-dimensional embeddings).

Future work directions include: (i) the experimentation with graph mining architectures such as graph neural networks and inductive representation learning methods (e.g., GraphSAGE [50]); (ii) the embedment of explainability features in the predictions provided by the proposed approach [51,52,53]; (iii) the exploitation of the rest of the information available in the scientific knowledge graph (e.g., the location, the whole text and the institution of the authors) [54,55,56].

## Figures and Tables

**Table 1:** Number of training samples (|Training subset samples|) and number of testing subset samples (|Testing subset samples|) of each dataset.

Dataset ID	|Training Subset Samples|	|Testing Subset Samples|
D1	1000	330
D2	1000	330
D3	1000	330
D4	1000	330
D5	1000	330
D6	1000	330
D7	6000	1890
D8		9900
D9	1000	330
D10	1000	330

**Table 2:** The features of each sample of the extracted datasets. A feature is associated with either a textual or a structural relationship of two authors.

Feature	Description	Type
adamic adar	The sum of the inverse logarithm of the degree of the set of common neighbor ‘*Author*’ nodes shared by a pair of nodes.	Structural (SF)
common neighbors	The number of neighbor ‘*Author*’ nodes that are common for a pair of ‘*Author*’ nodes.	Structural (SF)
preferential attachment	The product of the in-degree values of a pair of ‘*Author*’ nodes.	Structural (SF)
total neighbors	The product of the in-degree values of a pair of ‘*Author*’ nodes.	Structural (SF)
pyramid match	The similarity of the text of the graph-of-docs graphs of two nodes of ‘*Author*’ type using the Pyramid match graph kernel. The Propagation graph kernel incorporates the terms, the corresponding label of each term and the structure of the text into its formula, aiming to calculate the similarity between two texts.	Textual (TF)
propagation	The similarity of the text of the graph-of-docs graphs of two nodes of ‘*Author*’ type using the Propagation graph kernel. The Propagation graph kernel incorporates the terms, the corresponding word embedding of each term and the structure of the text into its formula, aiming to calculate the similarity between two texts.	Textual (TF)
weisfeiler pyramid match	The similarity of the text of the graph-of-docs graphs of two nodes of ‘*Author*’ type using the Weisfeiler Lehman framework and the Pyramid match graph kernel. The Weisfeiler Pyramid match graph kernel incorporates the terms, the corresponding label of each term and the structure of the text into its formula, aiming to calculate the similarity between two texts.	Textual (TF)
jaccard	The similarity of the text of the graph-of-docs graphs of two nodes of ‘*Author*’ type using the Jaccard coefficient. The Jaccard index deals only with the absence or the presence of a term into a text.	Structural and Textual (SF and TF)
Label	It denotes an edge of the ‘*co_authors*’ type between two nodes of the ‘*Author*’ type. A positive value (1) represents the existence, while a negative value (0) represents the absence of the edge.	Class

**Table 3:** The various features combinations in order to test how the different combinations affect the performance of the ML models in link prediction.

Feature Combination Name	Features Included	Proposed In
ALL	Adamic Adar, Common Neighbors, Preferential attachment, Total Neighbors, Pyramid match, Weisfeiler Pyramid match, Jaccard, Propagation	[8]
PM	Pyramid Match	[20]
WPM	Weisfeiler Pyramid match	[20]
AA_J (baseline)	Adamic Adar, Jaccard	[8]
AA (baseline)	Adamic Adar	[13]
*p*	Propagation	[21]
J (baseline)	Jaccard	[14,38]
AA_WPM	Adamic Adar, Weisfeiler Pyramid match	
AA_P	Adamic Adar, Propagation	
AA_PM	Adamic Adar, Pyramid match	

**Table 4:** Performance of the logistic regression classifier for each feature combination. * indicates statistical significance in improvement (*p* < 0.05) for each evaluation metric using the micro sign test against the AA_J baseline.

Feature Combination	Accuracy	Recall	Precision
ALL	0.6588	0.9963 *	0.6345
J	0.5093	0.0233	**1.0**
AA	0.9818	0.9643	0.9995
AA_J	0.9834	0.9671	0.9998
*p*	0.6669	0.5589	0.8157
PM	0.838	0.6965	0.9752
WPM	0.9476	0.9044	0.9905
AA_P	0.9652	0.9923 *	0.9625
AA_PM	0.998 *	0.9966 *	0.9995
AA_WPM	0.9986 *	0.9977 *	0.9995

**Table 5:** Performance of the neural network classifier for each feature combination. * indicates statistical significance in improvement (*p* < 0.05) for each evaluation metric using the micro sign test against the AA_J baseline. Average binary cross-entropy between real and predicted label value is considered as the train and test loss.

Feature Combination	Accuracy	Recall	Precision	Train Loss	Test Loss	Abs Loss Difference
ALL	0.9908	0.9931 *	0.9886	0.102	0.0499	0.0521
J	0.5093	0.0233	**1.0**	0.6647	0.6858	**0.0211**
AA	0.9922	0.985	0.9995	0.1303	0.0497	0.0806
AA_J	0.9925	0.9856	0.9995	0.1097	0.0413	0.0684
*p*	0.6954	0.5045	0.8624	0.6289	0.6057	0.0232
PM	0.8452	0.7085	0.9816	0.3219	0.399	0.0771
WPM	0.9248	0.859	0.9905	0.2612	0.239	0.0222
AA_P	0.9923	0.9851	0.9995	0.1311	0.0464	0.0847
AA_PM	0.994 *	0.9886 *	0.9995	0.1281	0.0395	0.0886
AA_WPM	0.9932	0.987	0.9995	0.1108	0.0372	0.0736

## Data Availability

The data are available at https://github.com/nkanak/cordkel (accessed on 25 April 2021).
